# Management of a Hypomineralisation of the Enamel by Applying a Remineraliser Based on Zinc Hydroxyapatite (microRepair)

**DOI:** 10.1155/2021/5291858

**Published:** 2021-12-16

**Authors:** G. Solinas, V. Grabesu, M. Lattari, R. Strinna, N. Arnould, A. A. Amodeo

**Affiliations:** ^1^RDH, Independent Researcher in Sassari, Italy; ^2^DDS and SLP, Independent Researcher in Sassari, Italy; ^3^RDH, Independent Researcher in Pavia, Italy; ^4^DDS, Independent Researcher in Sassari, Italy; ^5^RDH DHA, Adjunct Professor Degree Course Dental Hygiene University of Milan Department of Biomedical, Surgical and Dental Sciences, Italy; ^6^RDH DHA, Foundation IRCCS Ca' Granda Ospedale Maggiore Policlinico, Italy

## Abstract

According to our experience, the treatment with remineralising mousse based on biomimetic nanohydroxyapatite has the advantage of being easily implemented by all patients as it is economical and absolutely noninvasive. The following case report reports the results obtained from the use of a mousse based on biomimetic nanohydroxyapatite for the treatment of incisor and molar hypomineralisation. This case report illustrates the case of a 4-year-old patient who was diagnosed with MIH and was subjected to remineralising treatments at home for six months, at alternating periods. Throughout the observation period, the painful perception of the lesions was detected through an assessment scale, and the clinical appearance was documented photographically. One year after the diagnosis, all the elements involved no longer showed any symptoms.

## 1. Introduction

Incisor and molar hypomineralisation (MIH) is characterised by a defect in enamel development of systemic origin and affects at least one permanent first molar, often associated with enamel defects of the incisors [1].

MIH has a reported overall prevalence of 12.9% (11.7-14.3%) [2]. To date, the aetiology of this disease remains unclear. Several theories have developed over the years, including prenatal theories such as cigarette smoking or maternal infections, perinatal theories such as premature births, caesarean sections and complications during delivery, and postnatal theories such as breastfeeding, poor nutrition, childhood illnesses, and drug intake. None of these factors can be considered causal [3–5].

MIH elements show macroscopic clinical differences. Histologically, they show malposition of enamel crystals. Mechanically, they are more fragile and have reduced elasticity. Enamel is characterised by an increased amount of type I collagen, a1-antitrypsin, and antithrombin III, all of which inhibit the growth of hydroxyapatite crystals during enamel maturation.

Compared to healthy teeth, teeth affected by enamel defects show a less distinct histologically prism sheath, with a lack of hydroxyapatite crystals: the hypomineralised enamel has lower mechanical properties, such as hardness and elasticity, which have lower values than a normal enamel. In addition, the enamel affected by these pathologies shows a greater amount of proteins such as serum albumin, type I collagen, ameloblastin, a1-antitrypsin, and antitrobin III, which inhibit the growth of the hydroxyapatite crystals, resulting in the reduction of the enamel minerals [6].

The features for the diagnosis of MIH are shown in Table 1.

Recently, research is moving towards the introduction of remineralising material alternative to fluorine and based on the integration of calcium and phosphates at the level of demineralised dental surfaces [7].

Biomimetic effect of the nanohydroxyapatite is based on its interaction with biological tissues and on its ability to mimic biogenic materials in their functionalities. Chemical composition is similar to enamel and dentin, with intermediate characteristics. Gradual action of biomimetic nanohydroxyapatite crystals allows the linkage to dentinal and enamel tooth surfaces due to bioreabsorption properties under physiological conditions. This property can be modulated by ion substitution, and crystallinity degree achieved implementing innovative synthesis with nanosized crystal control [7, 8].

## 2. Differential Diagnosis

Particular emphasis should be placed on differential diagnosis with other abnormalities of the enamel structure. The correct diagnosis will result in a structured protocol for resolving the case. Specifically, we need to differentiate MIH from the following:

- Amelogenesis imperfecta: amelogenesis imperfecta is a genetic defect in enamel development and presents itself as hypoplastic, hypomatous or hypomineralised enamel depending on the stage of enamel formation affected by the congenital defect [9]. Depending on the type, it is complicated to distinguish it from a case of MIH. The family medical history and the involvement of the deciduous dentition should undoubtedly be taken into account [10]
- *Enamel hypoplasia:* this is characterised by a defect in the amount of enamel and presents itself with reduced enamel thickness, including cavities or irregular areas. Enamel loss is localised [11]. The differentiation from MIH should be based on the characteristics of the lesion edges, which are smoother and more regular in hypoplasia and more irregular and deeper in cases of MIH [10]
- *Fluorosis:* fluorosis is characterised by excessive fluoride absorption during the mineralisation phase of teeth [12]. The elements present with white, yellow or brown opacities but without a clear border with the enamel [9]. It is precisely the distinction of the lesions that is the decisive aspect in the differential diagnosis. The patient's medical history will provide additional help in making the diagnosis [10]
- *White spot lesions:* these are the first signs of carious lesions. They are caused by a prolonged accumulation of bacterial plaque on the tooth surface. The position of these lesions leads to differentiation from MIH, as they are most often found on cervical areas of the elements [10–13]

## 3. Materials and Methods

### 3.1. Timeline

**Figure 1:**
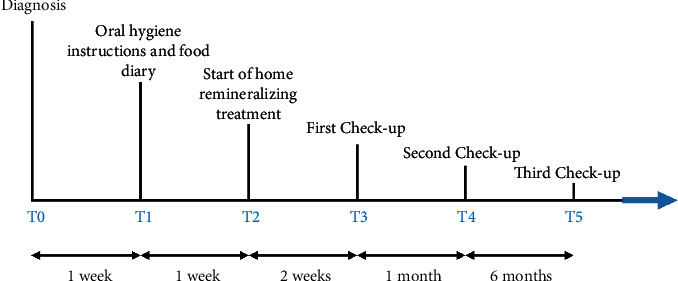
Timeline of interventions.

Figure 1 shows the timeline of the interventions. The timing of each intervention was agreed upon.

### 3.2. Patient Information

The female patient was 4 years old when she came to our attention. She was brought to the practice by her parents because she complained of hypersensitivity to heat and cold, even during normal breathing. The patient had never undergone a dental examination or treatment.

### 3.3. Objective Examination

During the examination, the patient presented hypomineralisation of the enamel on 11, 21, 31, 41, and 36. The appearance varies from one element to another. All the lesions on the incisors had well-defined borders and a white-yellow colour. 36 show areas of hypomineralisation at the occlusal and vestibular levels. The enamel is smooth on the incisors and broken on 36.

### 3.4. Diagnostic Evaluation

After careful assessment of the lesions during clinical follow-up, the diagnosis of MIH was made, evaluating the patient's medical history and creating a differential diagnosis with other enamel lesions.

There are currently several approaches to classifying the severity of MIH. In most cases, the pathology is subdivided and classified as mild or severe. The teeth show demarcated enamel opacities in mild cases without enamel breakdown and rarely hypersensitivity to external stimuli. In more severe cases, the enamel appears with demarcated opacities, enamel breakdown, and spontaneous hypersensitivity.

A new index, the MIH treatment need index (MIH-TNI), has recently been introduced (Table 2).

It is designed to classify MIH according to its severity based on two key symptoms that are clinically considered the most important: hypersensitivity and enamel breakdown [14–16].

For the diagnosis of our patient's MIH, we used the subdivision into sextants as proposed by the authors of the MIH-TNI [15]. The subdivision into sextants is shown in Table 3.

This clinical case was classified as shown in Table 4.

### 3.5. Interventions

Treatment options for MIH tooth range from prevention to extraction are shown. To make the right choice, several factors need to be taken into account:

- The severity of the condition
- Presence of symptoms
- Age of the patient
- Patient and parental compliance
- Patient and parental expectations [17]

The study by Williams et al. from 2006 [18] shows an initial clinical approach with six-step management:

1. Risk identification
2. Early diagnosis
3. Remineralisation and desensitisation
4. Prevention of tooth decay and enamel breakdown
5. Restorations or extractions
6. Maintenance

Also, in 2006, Mathu-Muju published another decision tree in the treatment of MIH [19], and Wright divided the injuries according to their severity (mild, moderate, and severe). Following this work, Lygidakis et al. published a practical guide in 2010 proposing various approaches depending on the severity and age of the patient [17].

**Figure 2:**
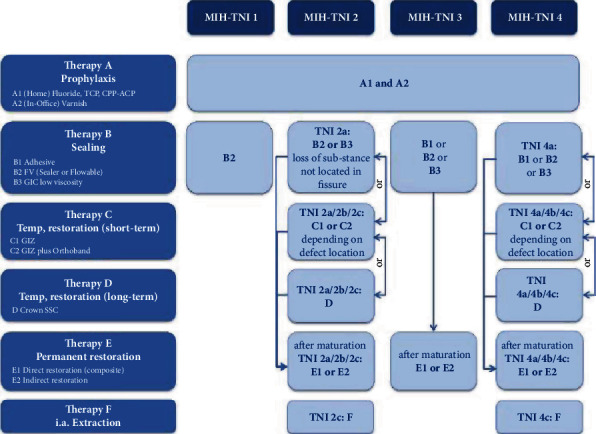
Therapy plan based on the MIH-TNI in patients with low caries risk.

**Figure 3:**
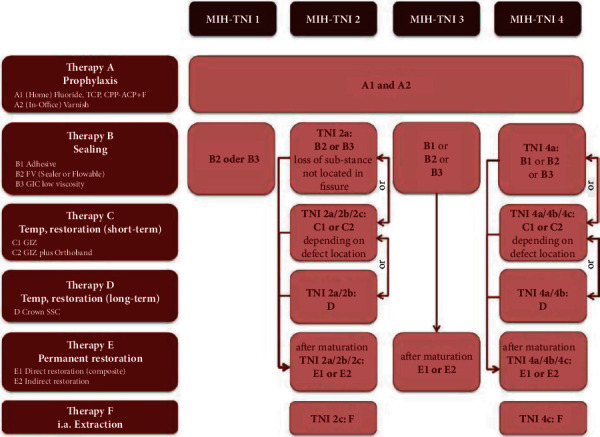
Therapy plan based on the MIH-TNI in patients with high caries risk.

By combining these guidelines and cross-referencing the data collected with the MIH-TNI table, taking the symptoms as a fixed point, therapeutic approaches ranging from prophylaxis to extraction have been developed [16] (Figures 2 and 3).

In this case report, we decided, in agreement with the patient's parents, to start with the least invasive treatment and then proceed gradually with increasingly invasive treatments in case of failure. Our treatment plan was exposed to the University's Internal Review Board which, with protocol 0077/2020, expressed a positive opinion.

**Figure 4:**
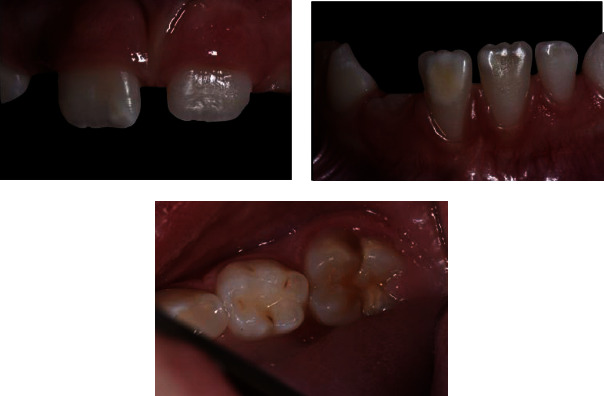
Photo of the patient at *T*0, i.e., at the time of diagnosis.

In this case, the first step (*T*0) was to confirm the diagnosis, collect the clinical data, and photographically document the initial stage (Figure 4). During the first appointment, informed consent was given and explained to the patient's parents.

Before drawing up the treatment plan, we had to classify the patient's perception of pain on each element that presented hypoplasia.

The cause of this hypersensitivity would appear to be chronic inflammation of the pulp. The hypomineralised enamel and dentine layers do not sufficiently protect the pulp tissue of the dental elements from chemical and physical stimuli and thermal stimuli in the oral cavity. Although a large part of the literature [1] states that hypersensitivity occurs almost exclusively on the first molars, we will see how the patient in question also reported an increased pain perception on other elements.

**Figure 5:**
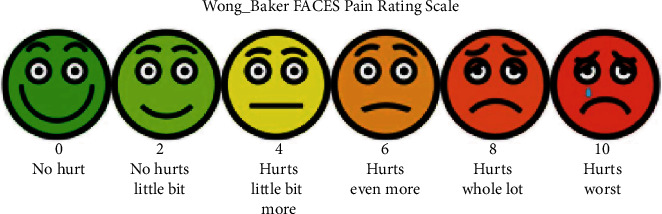
Wong-Baker face pain rating scale.

Given the patient's age, who was 4 years old at *T*0, we used the Wong-Baker face (Figure 5).

For a more reliable figure, considering the patient's age, we repeated the test three times at a distance of one week. The results are superimposable and specifically are as follows:

- Element 11 (perceived value 0): (no pain)
- Element 21 (perceived value 0): (no pain)
- Element 36 (perceived value 8): (very strong pain)
- Element 41 (perceived value 8): (very strong pain)

The possibility of a potential enamel fracture on 36 was then considered very remote, and for this reason, it was decided, at least in this first phase, not to subject the element to restorative treatment.

The next appointment (*T*1) served to instruct and motivate both the patient and the parent to maintain proper, almost obsessive, oral hygiene at home. We provided the necessary tools (electric toothbrush and plaque detector tablets).

**Figure 6:**
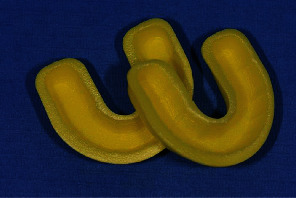
Disposable small fluoride trays.

At the next appointment (*T*2), one week later, we proceeded to start the home remineralisation therapy with the application of a paste with zinc hydroxyapatite (Biorepair® Repair Shock Treatment, Coswell, S.p.A., Funo, Italy; ingredients: aqua, zinc hydroxyapatite 30%, hydrated silica, silica, sodium myristoyl sarcosinate, sodium methyl cocoyl taurate, sodium bicarbonate, aroma, sodium saccharin, phenoxyethanol, benzyl alcohol, sodium benzoate, citric acid, and menthol) once a day for 5 minutes, using a small disposable tray for topical fluoroprophylaxis (Figure 6) Furthermore, for the patient's daily oral hygiene, it was required to use a specific toothpaste containing zinc hydroxyapatite (Biorepair Kids 0/6 years, Coswell, S.p.A., Funo, Italy; ingredients: aqua, glycerin, zinc hydroxyapatite^∗^, sorbitol, PEG-32, silica, cellulose gum, aroma, sodium myristoyl sarcosinate, sodium methyl cocoyl taurate, Fragaria vesca juice, Mentha piperita oil, sodium saccharin, citric acid, sodium benzoate, potassium sorbate, phenoxyethanol, benzyl alcohol, anethole, and menthol) under the supervision of the parents.

The toothpaste was used in a “pea size” dose, 2 minutes twice a day.

The parents were asked to check with the plaque detector once a week and take a photograph with their mobile phone. The pictures were sent to the practice to monitor the situation weekly without having to examine the patient so frequently.

The patient's food diary was collected and analysed, and there were no abnormalities in the weekly diet.

At time *T*3 (two weeks after the start of treatment), a clinical check-up was carried out with detection of pain perception:

- Element 11 (perceived value 0): (no pain)
- Element 21 (perceived value 0): (no pain)
- Element 36 (perceived value 8): (extreme pain)
- Element 41 (perceived value 8): (extreme pain)

The data collected showed no change from *T*0.

After one month (*T*4), a further clinical check-up was carried out with data collection and photo documentation:

- Element 11 (perceived value 0): (no pain)
- Element 21 (perceived value 0): (no pain)
- Element 36 (perceived value 5): (mild pain)
- Element 41 (perceived value 4): (mild pain)
- Element 32 (perceived value 9): (very strong pain)
- Element 42 (perceived value 2): (very slight pain)

Given the excellent compliance achieved by both patient and mother, the absence of side effects of the treatment and pain perception at time *T*4 showed a reduction in pain; we decided to extend the therapy for another 6 months.

This choice was also dictated by the fact that other elements, such as 32 and 42, started erupting and had enamel defects referable to MIH.

At this point, we started scheduling bimonthly check-ups in the studio.

**Figure 7:**
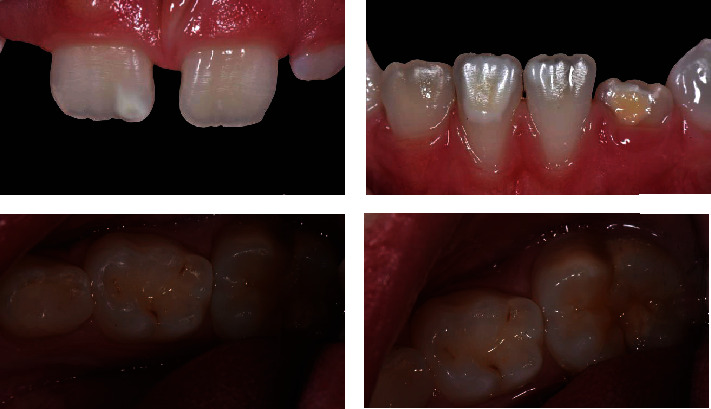
*T*5 photos (photos six months after the start of treatment).

We collected sensitivity data and documented the case photographically (Figure 7) and clinically.

The results of the test at time T5 were as follows:

- Element 11 (perceived value 0): (no pain)
- Element 21 (perceived value 0): (no pain)
- Element 36 (perceived value 0): (no pain)
- Element 41 (perceived value 0): (no pain)
- Element 32 (perceived value 6): (mild pain)
- Element 42 (perceived value 0): (no pain)

**Figure 8:**
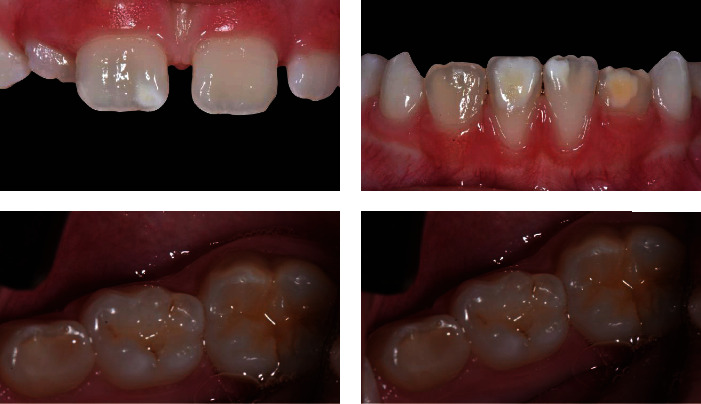
Photos at *T*6 (photos one year after the start of treatment before triage placement).

At 12 months after diagnosis (*T*6), we decided to safeguard the seal of the molars with the placement of glass ionomer cement (triage) as remineralisation of the elements after one year of treatment could guarantee stable adhesion of the product (Figure 8).

## 4. Discussion

This case report presents the results of home remineralisation treatment in a patient with MIH.

Incisor and molar hypomineralisation (MIH) can be defined as a delimited qualitative developmental defect of systemic origin in enamel involving the incisors and first permanent molars.

This condition leads to several dental complications, including hypersensitive teeth, increased susceptibility to tooth decay, impaired chewing due to rapid tooth loss, and aesthetic repercussions. These issues may affect patients' quality of life from an early age, so it is essential to diagnose these lesions early and set up treatment plans to preserve the teeth as much as possible. However, deciding on the most appropriate approach is complex. The main factors that need to be taken into account are patient compliance, stage of dental development, and severity of the defect; in addition, patient and parental preferences, other anomalies, and the psychosocial impact of treatment on the patient need to be considered [1].

In this case report, given the very young age of the patient and her compliance, a minimally invasive and as conservative as possible treatment was opted for.

One of the probable keys to our success was the patient's absolute cooperation. The remineralising paste was used for 5 minutes a day with a small disposable tray for topical fluoroprophylaxis; therefore, it was absolutely comfortable and atraumatic.

Several clinical trials [20–23] have investigated the benefits of using remineralising agents such as fluoride in its various formulations, casein-based mousses and amorphous calcium phosphate (CPP-ACP), and hydroxyapatite. Increasing the mineral component in teeth affected by MIH can improve the physical structure of the enamel, an improvement in symptoms and specifically in hypersensitivity, and an improvement in the aesthetic appearance of the teeth involved.

An important consideration which deserves to be done is the comparison of biomimetic hydroxyapatite with respect to fluoride.

Mouthwashes and toothpastes containing fluoride ions are among the most popular products for oral hygiene. Fluoride ions have the capability of interacting with dental hydroxyapatite crystals, thus forming the less insoluble fluoridated hydroxyapatite or fluorapatite, which are more resistant to the acid attack [24]. However, the efficacy of fluoride toothpastes is limited to a partial substitution of the hydroxyl groups with fluoride ions in natural hydroxyapatite, with no deposition of an additional mineral content.

Conversely, several studies [25] have shown that biomimetic hydroxyapatite even leads to a mineral deposition, thus forming a real coating on enamel and dental surfaces [26].

Additionally, the use of fluoride-based products may be linked to a risk of toxicity in the case of high dosage intake, respectively, consisting of fluorosis in children and bone diseases in the elderly.

The European Food Safety Authority (EFSA) suggests that the maximum level of fluoride content for oral care products, including toothpastes, is 1500 mg/kg. Also, the maximum fluoride intake should be 0.1 mg fluoride/kg/day in children aged 1–8 years [24].

Based on this consideration, in addition to the different remineralising effects, to lower the risk of toxicity linked to fluoride might suggest the safer use of biomimetic hydroxyapatite in young patients [27].

In our case report, one year after starting treatment with a hydroxyapatite paste, we can see that the probable increase in the mineral component has led to the almost complete resolution of hypersensitivity and improved aesthetic appearance of both the upper and lower central incisors. This encourages us to continue along this path in the hope that it will not be necessary to intervene in a more invasive manner in the future.

## 5. Conclusions

According to the latest scientific literature, MIH can significantly negatively impact children's lives, with hypersensitivity, increased susceptibility to caries, and repercussions on dental aesthetics among the most common symptoms.

Therefore, it is essential to know and distinguish MIH from other enamel lesions to intervene early and with a minimally invasive approach.

This case report reports our positive experience with treating a 4-year-old patient with stage 4b MIH using a hydroxyapatite-based cream (microRepair®). Within one year, the hypersensitivity was almost entirely resolved, and the aesthetics of the teeth improved.

## Figures and Tables

**Table 1:** Features for the diagnosis of MIH.

Key features	Description
Bounded opacity	(i) Delimited opacity (ii) Variability of colour and size (iii) Defects of less than 1 mm

Loss of posteructive enamel	(i) Surface defect after tooth eruption (ii) Loss of enamel from a surface initially formed after tooth eruption (iii) Frequently associated with a preexisting demarcated opacity

Atypical restorations	(i) Size and shape of restorations not in accordance with carious images (ii) Frequent extensions to buccal and palatal/lingual surfaces (iii) Frequently associated with opacity at the margin of the restoration (iv) For the incisors, buccal restoration not related to trauma can be seen

Molar extraction due to MIH	(i) The absence of a permanent first molar should be related to the other teeth (ii) Opacities and atypical restorations in other permanent first molars combined with the lack of a permanent first molar (iii) Absence of the first permanent molars in an otherwise healthy dentition in combination with demarcated opacities on the incisors

Failure of a molar or incisor to erupt	(i) The permanent first molar or incisor to be examined has not yet been erupted

**Table 2:** MIH treatment need index (MIH-THI).

Grade	Description
Grade 0	No MIH
Grade 1	MIH without hypersensitivity, without defect
Grade 2	MIH without hypersensitivity, with defect
2a	<1/3 of the area involved
2b	>1/3 of the area involved
2c	>2/3 of the involved surface and/or defect close to the pulp or atypical extraction or restoration
Grade 3	MIH with hypersensitivity, without defect
Grade 4	MIH with hypersensitivity, with defect
4a	<1/3 of the area involved
4b	>1/3 < 2/3 of the surface involved
4c	>2/3 of the involved surface and/or defect close to the pulp or atypical extraction or restoration

**Table 3:** Subdivision into sextants.

Sextant 1 Right maxilla distal to/with 14 (54).	Sextant 2 Anterior maxilla with 13-23 (53-63).	Sextant 3 Left maxilla distal to/with 24 (64).

Sextant 6 Right mandible distal to/with 44 (84).	Sextant 5 Anterior mandible with 33-43 (73-83).	Sextant 4 Left mandible distal to/with 34 (74).

**Table 4:** Clinical case classification.

Sextant 1 0	Sextant 2 2a	Sextant 3 0
Sextant 6 0	Sextant 5 4b	Sextant 4 4b
